# Detection of Erosive Changes on Smooth Surfaces with and without Orthodontic Brackets Using an Intraoral Scanner—An In Vitro Study

**DOI:** 10.3390/diagnostics13203232

**Published:** 2023-10-17

**Authors:** Anahita Jablonski-Momeni, Franka Hanselmann, Peter Bottenberg, Heike Korbmacher-Steiner

**Affiliations:** 1 Department of Orthodontics, Dental School, Medical Faculty, Philipps-University Marburg, 35033 Marburg, Germany; franka-hanselmann@t-online.de (F.H.); korbmacher@med.uni-marburg.de (H.K.-S.); 2 Department of Oral Health Care, Free University of Brussels (ULB-VUB), 1050 Brussels, Belgium; peter.bottenberg@ulb.be

**Keywords:** erosion, intraoral scanner, orthodontic brackets, soft drinks

## Abstract

Background: Consumption of acidic beverages favours development of erosions. Modern diagnostic methods are required to detect erosions at an early stage. This study aimed to evaluate the suitability of an intraoral scanner (IOS) for detection of erosive changes on smooth surfaces adjacent to orthodontic brackets. Methods: Orthodontic metal brackets were attached to the buccal surfaces of 58 extracted permanent human teeth. Teeth were randomly divided into groups: A = 6% citric acid, B = Coca-Cola, C = Redbull, D = Powerade, E = Control, no acid exposure. Teeth were exposed to acid in three erosion cycles, followed by rinsing and brushing. Scans of teeth were performed at baseline and after each erosion cycle and enamel loss was measured. Quantitative light-induced fluorescence (QLF) measurements were performed as reference standard. Results: Significant substance loss was measured in all acid groups after the second and third erosion cycle (*p* < 0.0001). Correlation between scans and QLF were significant (*p* = 0.001). Conclusions: With IOS, it was possible to detect and quantify enamel erosion at smooth surfaces with and without orthodontic brackets after a short exposure time. Considering the limitations of in vitro results, the use of IOS can be a promising digital tool to detect and monitor erosive enamel changes during fixed orthodontic treatment.

## 1. Introduction

Dental erosion is an irreversible loss of tooth structure caused by chemical processes unrelated to bacteria. In particular, the regular consumption of acidic foods can increase the risk of dental erosion [1,2]. Acidic beverages and foods like fizzy drinks and citrus fruits usually have a pH below the critical pH of 5.5 (for enamel) and 6.7 (for dentin) at which they can dissolve [3]. Typically, such beverages also have a high titratable acidity, which requires a longer time to neutralize [4]. Nowadays, high consumption of acidic beverages can be observed in adolescents and younger adults [5] which may contribute to dental erosion in adolescents. Moreover, high incidence and prevalence of enamel demineralization is reported in patients undergoing fixed orthodontic treatment with brackets [6].

The diagnosis of erosion is usually based on visual examination of clinical lesion features [7]. A new scoring system, the Basic Erosive Wear Examination (BEWE), was introduced to provide a simple tool for use in general practice and to allow comparison to other more discriminative indices [8]. The BEWE aimed to increase the awareness of tooth erosion amongst clinicians and general dental practitioners and to provide a guide as to its management.

The initial phase of enamel erosion is usually inconspicuous and not painful for the patient. Therefore, a diagnosis by optical tactile methods is not always possible due to the little pronounced clinical signs [9]. Only with more severe damage to the tooth structure does the macromorphology change, causing the tooth surface to appear silky smooth or, in exceptional cases, dull and matt. Progressive acid exposure can then lead to an uneven or stepped appearance of the surface, which in the further course can lead to dentine exposure and the complete loss of the morphology of the tooth [10]. Clinically, substance loss is perceptible only when a significant amount of enamel and possibly dentin has already been lost. Moreover, during orthodontic treatment, visual detection of dental erosion is hampered by the presence of oral biofilm and gingival hyperplasia. Therefore, in addition to visual examination, a number of other methods, such as quantitative light-induced fluorescence (QLF) or profilometry, have been investigated for the detection and quantification of tooth substance loss [11].

Such methods are often not suitable for everyday use in the dental office due to challenging application [12]. Due to the increasing prevalence of dental erosions [2], early detection and consequently early adoption of prophylactic measures is very important. Considering the digitalization in dentistry and the increasing use of intraoral scanners, there have been initial considerations to apply them also for the detection of erosions.

In vitro studies on bovine enamel already suggested an intraoral scanner as a potential clinical tool for detecting and quantitatively monitoring early and advanced erosive tooth wear [13]. Laboratory studies on human enamel also showed that intraoral scanners were able to detect small amounts of tissue loss under simulated clinical conditions [14]. Michou et al. [15] detected significant tooth substance loss with an intraoral scanner after 3 h of an erosive–abrasive challenge. Long-term clinical investigations showed that with intraoral scanner it was possible to display the progression of erosions in young adults reliably [16,17].

Still, there are no published studies yet that have investigated the use of intraoral scanners to measure initial erosive enamel loss adjacent to orthodontic brackets. Therefore, the present in vitro study aimed to evaluate the ability of an intraoral scanner (IOS, 3Shape TRIOS^®^ 4, Copenhagen, Denmark) to detect and quantify early erosive changes on smooth surfaces with and without orthodontic brackets.

## 2. Materials and Methods

### 2.1. Sample Selection and Preparation

Extracted human permanent teeth were used in this in vitro study. Informed consent prior to extraction was obtained. The approval of the Ethics Committee of the Medical faculty of Philipps University of Marburg was available for use of extracted teeth for the in vitro study (file number 132/19). Teeth with visible enamel defects or carious lesions were excluded from the study. All teeth available for selection were cleaned using scalers and brushes (miniature tooth cleaning brushes, Pluradent, Offenbach, Germany) and stored in 0.001% sodium azide solution for disinfection at the beginning of the study. A sample size calculation was performed based on preliminary investigations (MedCalc Statistical Software, version 20.010, Ostend, Belgium). With a significance level of α = 0.05 and a power of 95%; at least 25 samples should be included. A total of 58 teeth were randomly divided into five groups (*n* = 12 groups A to D, *n* = 10 teeth group E). The respective acid was then assigned to the groups:

A = 6% citric acid/pH value 1.6;
B = Coca-Cola/pH value 2.6;
C = Redbull/pH value 3.6;
D = Powerade/pH-value 3.9;
E = Control group, deionized water/pH-value 7.0.

**Figure 1:**
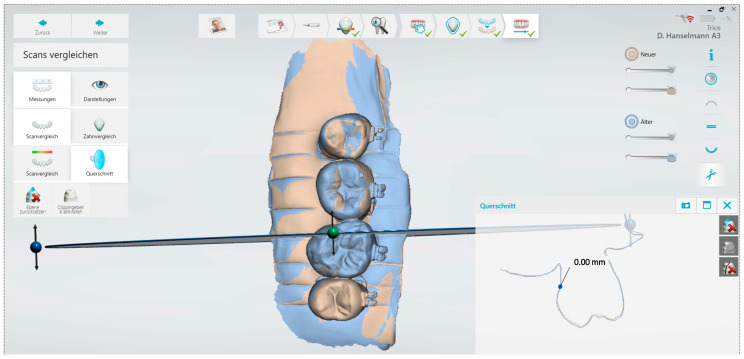
Scan of a sample (#A10) with markers for simplified overlaying of the scans.

Subsequently, the teeth were embedded in silicone (betasil Vario Putty, Müller-Omicron GmbH & Co. KG, Lindlar, Germany) and an orthodontic bracket (Equilibrium mini, Dentaurum, Ispringen, Germany) was adhesively attached to each buccal surface. The enamel surface was etched (36% phosphoric acid gel: Conditioner 36, Dentsply DeTrey, Konstanz, Germany) and rinsed with water after 30 s. The teeth were dried, and orthodontic brackets were bonded in the centre of each sample (Transbond XT primer and adhesive, 3M Unitek, Landsberg, Germany) and were light cured (FlashMax P4 Ortho Pro, orthodontic light pen, CMS Dental, Copenhagen, Denmark). To ensure subsequent reproducibility of the measurements, markings in shape of lines were placed into the silicone (Figure 1). The samples were stored in deionized water between the erosion cycles.

### 2.2. Measurement with Intraoral Scanner and Acid Exposure

To document the initial condition, samples were scanned prior to fixing the brackets with the 3Shape TRIOS 4^®^ intraoral scanner (hereafter IOS) and the corresponding software (version 21.4.0, Copenhagen, Denmark) according to manufacturer’s recommendations. Then, brackets were inserted, and baseline scans (T0) were performed.

The samples were exposed to the erosive agents in three successive cycles. In each erosion cycle, the teeth were stirred manually for four minutes in a container with 200 mL of the respective solution. They were then rinsed with deionized water for one minute and manually brushed with a soft toothbrush (Oral-B Ortho toothbrush) for two minutes. The adhesion of the bracket to the tooth surface was controlled with a ball-ended probe after each cycle to ensure that the brackets had not become detached. After each of the three erosion cycles (T1, T2, T3), a scan with the IOS took place on carefully dried samples. To check the reproducibility of the measurements, the samples were scanned again (T3-2) after the third erosion cycle (T3-1). 

The 3D scans obtained after different cycles were overlaid with the baseline scan using the scanner’s internal software (version 21.4.0). As reference points, the surface of the brackets was manually marked on each scan using a brush tool. The marked surfaces were then used by the software as a constant reference for overlaying the 3D models. To record the substance loss of the samples after each erosion cycle compared to the baseline scan, the removal was measured on each tooth using a cross-section tool available in the software. This tool allowed very slight profile difference measurements in the micrometer range to be made on the 3D models to quantify tooth substance loss. The measurements took place using the markings vestibular, distal, and mesial of the bracket in each case, as well as lingual on the surface without bracket. The measurements were made adjacent to the brackets, as well as on the plain lingual surface without orthodontic bracket. The difference was measured as loss of enamel in µm.

### 2.3. Quantitative Light-Induced Fluorescence (QLF)

After each scan, surface analysis was performed using non-invasive measurements with QLF (Q-Ray Cam, Inspektor Research Systems BV, Amsterdam, The Netherlands). The Software C4 QLF Research Suite (version 1.08, Inspektor Research Systems BV, Amsterdam, The Netherlands) was used to quantify the fluorescence behaviour of the enamel surfaces which is directly proportional to the mineral content. Using the new QLF device, the fluorescence value of a sound surface is determined automatically by the software. Fluorescence loss in % (ΔF) was measured which correlates with lesion depth.

### 2.4. Statistical Analysis

Statistical analysis was performed with MedCalc software. Data were tested for normal distribution using the Shapiro–Wilk test (*p* < 0.01). Intraclass correlation coefficient was calculated for reproducibility of IOS measurements. Group comparison was performed using non-parametric tests (Kruskal–Wallis test). The measurements on surfaces with and without brackets were compared in each group with a *t*-test. Correlation between scans and QLF measurements were calculated by Spearman’s rank correlation coefficient (rs). The significance level was set at α = 0.05.

## 3. Results

In total, 58 investigation sites were measured on surfaces without brackets (lingual surfaces) and 116 sites were measured on the buccal surfaces, mesial and distal of each orthodontic bracket (Br).

**Figure 2:**
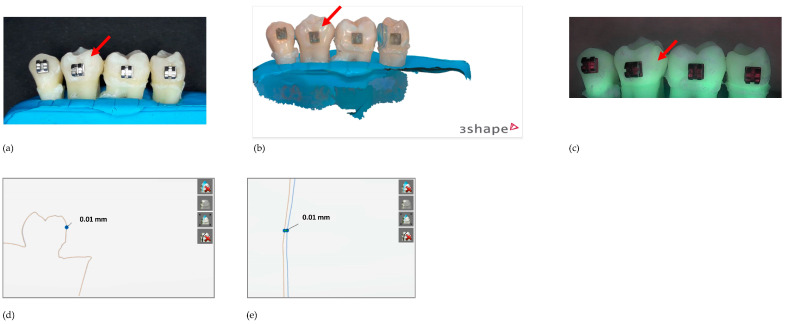
Sample #A10 after the first erosion cycle. The arrow indicates the surface of interest: (**a**) regular image, (**b**) scan, (**c**) QLF image, (**d**) overlay of scans T0_T1 = 10 µm, (**e**) zoom of the overlay of scans.

**Figure 3:**
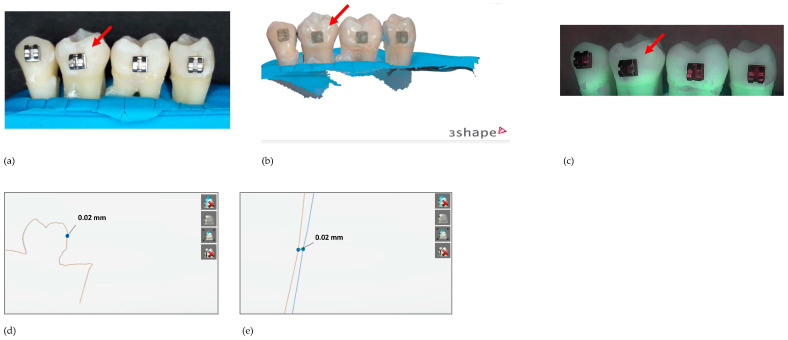
Same sample as in Figure 1 after the second erosion cycle. The arrow indicates the surface of interest: (**a**) regular image, (**b**) scan, (**c**) QLF image, (**d**) overlay of scans T0_T2 = 20 µm, (**e**) zoom of the overlay of scans.

**Figure 4:**
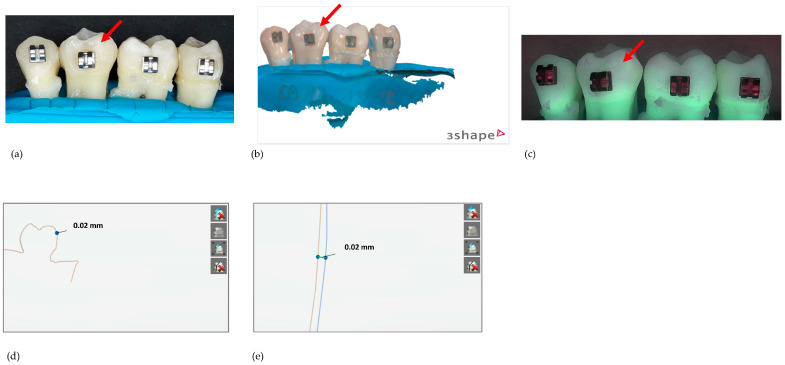
Same sample as in Figure 1 after the third erosion cycle. The arrow indicates the surface of interest: (**a**) regular image, (**b**) scan, (**c**) QLF image, (**d**) overlay of scans T0_T3 = 20 µm, (**e**) zoom of the overlay of scans.

Figure 2, Figure 3 and Figure 4 show examples of the teeth from a sample in group A (6% citric acid) with the corresponding scans and QLF images at different exposure times.

Intraclass correlation coefficient for the reproducibility of the scans was 0.94 (95% confidence interval: 0.90–0.96). After the first erosion cycle (T0_T1), the median substance removal was 0 µm for all groups (Table 1). After the second erosion cycle (T0_T2), a median enamel erosion of 10 µm was measured in groups A, ABr, and BBr. In groups C and D, a median substance erosion could only be measured after the third erosion cycle (T0_T3). The highest median enamel removal after the last erosion cycle was measured in groups A (6% citric acid, 12.5 µm) and B (Coca-Cola, 15 µm). In group E (control group), no enamel loss was observed. While no significant enamel loss was evident in the group comparison after the first erosion cycle, significant enamel erosion was measured by IOS after the second and third erosion cycles (*p* < 0.0001).

Regarding substance loss within the groups at the different erosion times, there was an increase in the measured loss of enamel over the course of the three erosion cycles in all groups except the control group. 

Comparison of scans of surfaces with and without brackets revealed no significant differences: *p*-values at T0_T1 = 0.94, at T0_T2 = 0.10, and at T0_T3: 0.64.

In comparing the QLF data at the different acid exposure times (Table 2), an increase in fluorescence loss was seen in all test groups. There was a significant negative correlation between the QLF and scan measurements from the first erosion cycle onwards (rs −0.16 to −0.44, *p* = 0.001).

## 4. Discussion

In the present study, the of ability of an intraoral scanner was evaluated for the first time to detect initial erosive changes on smooth surfaces with orthodontic brackets. The results showed that with the IOS, erosive tooth wear caused by different acids was measurable. The substance loss could be quantified on surfaces with and without orthodontic brackets in the same manner. Overall, it was possible to show even slight substance loss with the scanner, where the changes would not be detected visually (up to 20 µm). The limitations are most obviously the in vitro situation in which imaging could be performed in perfect conditions of exposure geometry and without the presence of a salivary film and biofilm. Another limitation of IOS is that a possible substance loss in enamel can only be measured by comparing at least two scans and excluding artifacts by exposure or image processing errors [18]. In the clinical situation, it is more convenient to look at the current situation of the affected tooth surfaces by means of further parameters such as surface structure or surfaces gloss and to diagnose possible erosion.

In view of the changed lifestyle with increased intake of acidic foods, especially in young adults, erosion-related tooth structure loss is of increasing clinical importance [2]. However, the loss of substance often only becomes clinically visible with advanced enamel loss. Therefore, in addition to awareness of the aetiology, early detection and follow-up of erosions is of great importance in order to initiate appropriate preventive and therapeutic measures. Especially in adolescents, who are at increased risk of enamel demineralization due to a fixed orthodontic appliance, a reproducible method for the diagnosis and early detection of dental erosions is essential.

The diagnosis of initial stages of erosive tooth wear is difficult to perform [19,20], and this limitation might be the first barrier for the correct detection and monitoring of lesion progression. Therefore, modern and chairside tools are required which enable to improve detection and documentation of erosions [21]. Up-to-date diagnostic tools such as the BEWE index have been recommended for the recording of erosive tooth wear in cross-sectional studies and at the dental practice [22]. However, the criteria description of BEWE provides general information, and therefore it may not be best suitable for progression studies of erosion, where detailed information is needed [8]. Nevertheless, the use of this index combined with additional records such as photographs, cast models, or 3D images provided by intra-oral scanners could improve its performance for the monitoring of erosive tooth wear [23]. 

Intraoral scanners are increasingly used in the field of diagnostics in addition to their original purpose in restorative dentistry. Recent studies have shown that the intraoral scanner is also suitable for the diagnosis and monitoring of erosion. As an example, the TRIOS^®^3 intraoral scanner and the associated software (version 21.4.0) were able to diagnose tooth structure loss at an early stage [15]. The integrated software makes it possible to overlay digital data sets that were acquired at different times. This overlay can either be generated manually by a three-point match or it is performed automatically by the software using a best-fit algorithm. 

It was investigated if quantitative analysis of intraoral scans of study models can identify erosive tooth wear progression. Data were collected from a retrospective longitudinal study, using pre-and post-orthodontic treatment casts of 11–13-year-olds at two appointments. The casts were digitized with an intraoral scanner. Based on the results, the quantitative analysis of digital technologies would be suitable as an adjunct tool to be used alongside history taking and clinical judgement of tooth wear [24]. Further investigations showed that the Basic Erosive Tooth Wear Index (BEWE index) is a suitable tool for the scoring of erosive tooth wear lesions in 3D images and casts [23]. Based on those results, it was suggested that the combination of both digital 3D records and index can be used for the monitoring of erosive tooth wear in a longitudinal approach [23]. Also, in other studies, models were scanned to observe the progression of erosion [25,26]. It should be mentioned that dimensional changes in the materials, such as the setting expansion of the gypsum, could affect the accuracy of measurements. In another study, substance loss of about 15 µm could be reliably detected with IOS [14]. In that study, the scan results were compared with profilometric measurements and showed good agreement.

Adolescents are a typical age group in which fixed orthodontic treatment may be necessary. Without appropriate prevention strategies, patients with fixed orthodontic treatment usually develop initial caries lesions [6]. The frequent consumption of acidic drinks is another risk factor for developing demineralization in addition to a cariogenic diet and this group is in the focus of developing erosions. This shows the relevance of the present study to detect erosive changes on smooth surfaces with orthodontic brackets. While some areas cannot always be assessed clinically or not sufficiently well, the advantage of an intraoral scanner is that all surfaces can be recorded and viewed afterwards. This would emphasize the benefit of our results.

In this study, the presence of brackets did not influence the erosion of the dental surface. The buccal and oral surfaces were exposed to the respective solution at the same time. Clinically, the loss of tooth structure associated with erosion can be observed on the facial, occlusal, and oral tooth surfaces with varying degrees of severity, depending on the etiology of the lesion. Intrinsic acid typically results in wear of the maxillary palatal surface ‘perimolysis’ and occlusal–lingual aspect of first mandibular molar teeth [27], whereas extrinsic acid is more associated with wear of the labial aspects of teeth. In a systematic review, varying degrees of tooth wear were reported after comprehensive orthodontic treatment [28]. The authors concluded that further studies are needed in order to evaluate how much is associated with orthodontic treatment and/or physiologic alterations of the dentition. In contrast, in a retrospective study, no significant differences in tooth wear in relation to need for or receipt of orthodontic treatment were recorded. Likewise, there appeared to be no significant association between tooth wear and reported intake of acidic drinks and foods [29]. Nevertheless, our clinical observation is that adolescents with brackets are at risk for developing erosions, in addition to the increased risk of caries, among other things because of their dietary and drinking habits. Therefore, teeth with brackets were the focus of our study.

QLF has been proven in several studies to quantify demineralization as a non-invasive reference standard [30,31,32]. The method is based on the different absorbance or reflectance of sound compared to demineralized tooth structure, where demineralization is associated with a loss of fluorescence. In the present study, we used the latest version of QLF where an additional control group is not necessary, since the reference of a surface (sound surface value) is determined automatically by the integrated software [30]. However, all measurements were performed prior to erosion as well as an internal control. The fluorescence behaviour before the erosion cycles was 0% for all samples in each group, indicating no other demineralization at baseline. The correlation values between the measured fluorescence loss by QLF and the measured substance loss by intraoral scanner showed that measurements with the IOS using the reference standard QLF were complementary. The substance loss measured by intraoral scanner and the fluorescence loss measured by QLF during the erosion cycles were opposite, reflected in positive µm values measured by intraoral scanner and in negative ∆F values measured by QLF. Studies on the relationship between fluorescence loss and depth of demineralization of enamel with erosion showed that QLF was capable of detecting and monitoring mineral loss in the enamel erosion model [33]. The fluorescence loss measured by QLF reflected not the depth of net tissue loss but the depth or mineral content of the decalcified region beneath the area of erosive tissue loss. Another method for examining tooth structure loss is profilometry. Since profilometry achieves the highest sensitivity on flat, polished surfaces [34], it is rarely used clinically. Next to a net material loss, as measured by IOS, acid exposure also produces a sub-surface mineral loss preceding the acid damage as demonstrated by QLF.

There were recent investigations in which it was shown that it was possible to combine a near-infrared device with an intraoral scanner [35]. If this experimental method can be clinically useable (at present, only laboratory evidence exists), it may enable practitioners to monitor surface and subsurface mineral loss with one device. A combination of clinical observation methods that are less dependent on operator appreciation. The more that the presence of orthodontic appliances can obfuscate the observer’s visual appreciation. Using indices like BEWE or ICDAS also are less discriminative for subtle changes when compared to a measurement method that allows obtaining numerical results when used in a follow-up setting.

Factors such as individual drinking behaviour influence the development of erosions [36]. In the present study, the highest enamel loss measured by intraoral scanner was caused by citric acid and Coca-Cola. The increase in substance loss within the groups from the first to the third erosion cycle could also be quantified by intraoral scanner. This makes it clear that the intraoral scanner can reliably detect and monitor progressive tooth substance loss. Initial studies regarding the digital detection and visualization of erosions using intraoral scanners provided good results [15,16]. One study showed that the frequency of acid exposure correlates with the measured substance erosion by intraoral scanner and thus monitoring of erosive tooth structure loss by intraoral scanner is possible [15]. In addition to the early detection of erosions, the present study also aimed to differentiate between enamel loss caused by different acidic soft drinks. Commercially available drinks that are frequently consumed in everyday life, especially by young adults, were selected [37,38]. In a study by Karda et al. [39], the high erosive potential of Coca-Cola was demonstrated. Furthermore, soft drinks such as Powerade were shown to cause more pronounced erosive changes in contrast to other isotonic drinks, which could be attributed to the low calcium content in Powerade [40].

Acidic drinks may affect the bond strength of brackets on the enamel surface or produce loss of adhesive and microleakage [41,42]. One study evaluated the effect of two soft drinks on bracket bond strength [43]. Samples which were exposed to soft drinks showed significantly lower resistance to shearing forces compared to those surfaces which were stored in artificial saliva control group. In the soft drink groups, areas of damage next to the adhesive were observed, which were typically caused by the acid [43]. In our study, we controlled the adhesion of the bracket to the tooth surface with a dental explorer, but no further shear bond strength was tested. This is subject to further investigation.

Overall, it is clear that there has been an increasing tendency in recent years to use digital technologies in the oral and dental health of children and adolescents [44]. Occlusal caries can also be detected on digital 3D images of permanent teeth scanned by intraoral scanners [45]. Digital imaging using intraoral scanners allows the practitioner to visualize the loss of tooth structure even in the early stages of dental erosion. Especially among young people, who have a high affinity for digital media, diagnostics by means of intraoral scanners could meet with a high level of acceptance. 

Although the results of in vitro studies cannot be transferred to the clinical situation without restrictions, the results presented form a good basis for further studies. Considering the in vitro design of the study, further clinical research is required in this field. By early detection and monitoring of erosive changes, etiological factors leading to erosion can be identified at the initial stage and ideally reduced or eliminated.

## 5. Conclusions and Clinical Relevance

In the present study, it was shown that initial erosive tooth substance loss caused by acid solutions on smooth surfaces with and without orthodontic brackets could already be recorded and quantified using intraoral scanners. The presence of metal brackets did not influence the scans, and the fluorescence-based measurements were affected. In addition, a correlation between the frequency of acid exposure and increasing substance loss was shown. Based on the results of the present study, the use of intraoral scanners can be recommended as a modern digital procedure during orthodontic treatment with fixed appliances. 

## Figures and Tables

**Table 1:** Comparison of baseline scans with scans after different erosion cycles (µm).

Group	Min-Max (T0_T1)/µm	Median (T0_T1)/µm	Min-Max (T0_T2)/µm	Median (T0_T2)/µm	Min-Max (T0_T2)/µm	Median (T0_T2)/µm
A	0–5	0	0–15	10	10–20	12.5
A Bracket	0–10	0	0–20	10	0–20	10
B	0–5	0	0–15	10	0–20	15
B Bracket	0–10	0	0–10	0	0–20	10
C	0	0	0–5	0	0–15	7.5
C Bracket	0	0	0–10	0	0–10	0
D	0–5	0	0–5	0	0–10	5
D Bracket	0	0	0–10	0	0–10	0
E	0	0	0	0	0	0
E Bracket	0	0	0	0	0	0

**Table 2:** QLF measurements after different erosion cycles (ΔF, %).

Group	Min-Max ΔF T1 (%)	Median ΔF T1 (%)	Min-Max ΔF T2 (%)	Median ΔF T2 (%)	Min-Max ΔF T3 (%)	Median ΔF T3 (%)
A	−8.6–0	−5.6	−16.7–0	−2.5	−11.6–0	−6.2
A Bracket	−12.7–0	0	−10.6–0	−3.0	−22.0–0	−5.9
B	−20.1–0	−5.6	−24.9–0	−6.0	−16.5–0	−6.1
B Bracket	−15.3–0	0	−16.6–0	0	−20.1–0	0
C	−13.2–0	−5.9	−17.9–0	−6.1	−12.4–0	−5.8
C Bracket	−11.8–0	−5.3	−15.3–0	−5.7	−10.3–0	0
D	−10.0–0	−6.8	−9.1–0	−7.1	−9.3–0	−7.0
D Bracket	−8.7–0	0	−9.6–0	0	−8.1–0	0
E	−13.1–0	0	−5.8–0	0	−5.6–0	0
E Bracket	0	0	0	0	0	0

## Data Availability

All data generated or analysed during this study are included in this article. Further inquiries can be directed to the corresponding author.
